# Synergistic cytotoxic effect of sulindac and pyrrolidine dithiocarbamate against ovarian cancer cells

**DOI:** 10.3892/or.2012.1639

**Published:** 2012-01-16

**Authors:** ANNA JAKUBOWSKA-MUĆKA, JACEK SIEŃKO, ŁUKASZ ZAPAŁA, RAFAŁ WOLNY, WITOLD LASEK

**Affiliations:** 1 Department of Immunology, Maria Skłodowska-Curie Memorial Cancer Center and Institute of Oncology, Warsaw; 2 Second Clinic of Obstetrics and Gynecology, Medical University of Warsaw, Warsaw; 3 Department of Immunology, Centre of Biostructure, Medical University of Warsaw, Warsaw, Poland

**Keywords:** sulindac, pyrrolidine dithiocarbamate, ovarian cancer, apoptosis

## Abstract

Sulindac, a non-steroidal anti-inflammatory drug, suppresses carcinogenesis and inhibits growth of tumor cells. Pyrrolidine dithiocarbamate (PDTC), a potent NF-κB inhibitor, has been also identified as a potential anti-neoplastic agent. We hypothesized that combination of sulindac and PDTC could result in augmentation of cytotoxicity against ovarian cancer cells. The effect of sulindac and PDTC was examined on several ovarian cancer lines. Tumor cell viability was assessed using the MTT assay. Annexin-V/PI staining was used to detect apoptosis, cell cycle distribution was analyzed in FACS, and expression of cellular proteins was detected by Western blotting. Incubation of OVA-14, OVP-10 and CAOV-1 ovarian cancer cells with sulindac and PDTC resulted in significantly greater inhibition of cell viability compared to either compound alone. In a model of OVA-14 cells it was evident that this effect was not related to the expression of COX enzymes since both active (sulindac sulfide) and inactive (sulindac) *in vitro* compounds affected the growth of tumor cells to a similar extent and synergized in cytotoxicity with PDTC. Combination of sulindac and PDTC lead to G0 arrest and massive apoptosis in co-treated cultures. Western blotting analysis argued for induction of the mitochondrial apoptotic pathway. These data demonstrate the synergistic cytotoxic effect of sulindac and PDTC on ovarian cancer cells through apoptosis and cell cycle arrest and prompt to test the efficacy of this combination in animal models.

## Introduction

Ovarian cancer is the most deadly malignancy of the female reproductive system with more than 70% of cases diagnosed at an advanced stage. Current treatment of this cancer includes cytoreductive surgery (tumor debulking) followed by platinum- and taxane-based therapy (1). However, despite a high rate of initial remissions, patients usually relapse and subsequently require additional second- or third-line therapy (2,3). Unfortunately, the patients eventually develop drug resistance, causing limitations of further treatment options and decreasing overall survival (1). Thus, intervention with chemopreventive agents or new adjuvant therapy may offer a desirable option for ovarian cancer (4).

Experimental studies, animal tumor models and many *in vitro* experiments have all demonstrated that non-steroidal anti-inflammatory drugs (NSAIDs) appear to be effective in chemoprevention and possible treatment of various types of cancer (5–10). The rationale of their use is their cyclooxygenases (COXs)-blocking activity. Overexpression of COX genes is a frequent phenomenon in preneoplastic and tumor tissues, including ovarian cancer (9), and is recognized as a bad prognostic factor. Upregulation of the COX enzymes in ovarian tumor cells has been implicated in platinum drug resistance and promotion of tumor progression (11,12). However, some studies suggest that overexpression of COXs is not obligatory for anticancer effect, at least in therapeutic approach, and NSAIDs can directly kill tumor cells via different intracellular pathways including NF-κB inhibition (10,13,14).

One of the most promising pharmaceutical agent from the group of NSAIDs, reported to inhibit carcinogenesis and acting directly against tumor cells *in vitro* and in experimental tumor models, is sulindac. This agent itself does not inhibit cyclooxygenases but is metabolized to COX-inhibiting sulindac sulfide and inactive sulindac sulfone. Sulindac and its derivatives, alone or in combination with some chemotherapeutics, have been found to induce growth suppression and apoptosis in cultures of tumor cells (15–22), including ovarian cancer (23). At present, sulindac is being evaluated as a chemopreventive or therapeutic agent in several clinical trials (NCT00755976, NCT00299195 and NCT00118365 available at http:/www.clinicaltrials.gov). There are also attempts to use sulindac sulfone, known as exisulind, in combination treatments of various types of cancer (24,25).

Pyrrolidine dithiocarbamate (PDTC) is a thiol-containing synthetic compound, which is known for its antioxidant, metal-chelating and strong NF-κB inhibitory properties (26,27). Occasionally, it exerts paradoxical prooxidant activity (28). Recently, PDTC has attracted the attention of investigators as a potential anticancer agent. In *in vitro* studies this agent exerted cytotoxic effects against many types of cancer cells (28–30). Interestingly from the therapeutic point of view, PDTC has been shown to inhibit blood vessel formation and tumor angiogenesis in *ex vivo* studies and in animal models (31).

In a previous study, we have demonstrated that sulindac and sulindac sulfide but not other NSAIDs such as acetylsalicylic acid and rofecoxib inhibited the growth of various ovarian cancer cells (32). We supposed that this effect could result from NF-κB targeting. The aim of the present study was: i) to assess the effect of sulindac or sulindac sulfide in combination with PDTC on the growth of cells of different ovarian cancer lines; and ii) to identify possible mechanisms of their action.

## Materials and methods

### Cell cultures

The following ovarian cancer cell lines were used in the study: OVA-14 (established in our laboratory from solid epithelial (serous) tumor, CAOV-1 (obtained from Dr M. Siedlar, Jagiellonian University Collegium Medicum, Krakow), OVP-10 (obtained from Dr B. Szaniawska, Institute of Oncology, Warsaw), MDAH 2774 (ATCC no. CRL-10303), SKOV-3 (ATCC no. HTB-77). The cells were grown in Dulbecco’s modified Eagle’s medium (DMEM, Gibco-BRL, Invitrogen) (OVA-14, CAOV-1, MDAH 2774, SKOV-4) or RPMI-1640 (Gibco-BRL, Invitrogen) (OVP-10) supplemented with 10% heat-inactivated fetal calf serum (FCS, Gibco-BRL, Invitrogen) and antibiotic-antimycotic (Sigma). The cells were maintained in 25-cm^2^ tissue flasks (Nunc, Roskdile, Denmark) at 37°C in a humidified atmosphere of 5% CO_2_ and were passaged two to three times weekly.

### Drugs

Sulindac was from Sigma and sulindac sulfide was purchased from Biomol Research Laboratories (Plymouth Meeting, PA, USA). The drugs were dissolved in dimethylsulfoxide (DMSO; Sigma) and stock solutions were prepared (200 mM) for further preparation. The cells in control cultures were incubated either without diluent or with 0.1% DMSO; no significant differences in cell growth were observed. Pyrrolidine dithiocarbamate (PDTC) was purchased from Sigma, with the stock solution (200 mM) prepared in distilled water.

### MTT assay

The cytotoxic effects of sulindac and/or PDTC on ovarian cancer cells was tested in a standard 3-(4,5-dimethylthiazol-2-yl)-2,5-diphenyltetrazolium bromide (MTT) assay. This assay relies on the ability of viable cells to reduce a yellow MTT to a purple formazan product. Cells were incubated in 96-well plates (2×10^4^/200 μl/well) with PDTC (final concentrations: 1, 2, 4, 8 and 16 μM) and sulindac or sulindac sulfide (final concentrations: 50, 100 and 200 μM), alone or in combination, for 24 h. At the end of the incubation, 25 μl of MTT (2.5 mg/ml) was added to each well for the last 4 h. Then the cells were centrifuged (350 × g, 10 min), supernatants were removed and the formazan product was dissolved in acid DMSO. The plates were read on an ELISA reader (SLT-Labinstruments, Salzburg, Austria) using a 550 nm filter. The means and standard deviations were determined for triplicate samples. The cytotoxic effect was expressed as the relative viability and was calculated as follows: relative viability = [(experimental absorbance - background absorbance)/(absorbance of vehicle-treated cells - background absorbance) × 100.

### Western blot analysis

At the end of incubation with sulindac ± PDTC (1 or 4 h), OVA-14 cells were washed three times in PBS and were lysed with RIPA buffer containing protease inhibitor cocktail (PMSF 0.1 mg/ml, aprotinin 1.7 mg/ml, pepstatin 5 μg/ml, leupeptin 5 mg/ml, sodium orthovanadate 1 mM) (protease inhibitor cocktail:RIPA buffer, 1:100). The protein concentration in the lysates was determined by Lowry’s method. The cell extract was separated by 10% gel (NF-κB p50, NF-κB p65, GAPDH) or by 12% gel (Bcl-2, Bax, procaspase-9) and transferred onto a nitrocellulose membrane (Bio-Rad Laboratories). The membrane was blocked with 5% non-fat milk in TBST (25 mM Tris, pH 7.6, 138 mM NaCl and 0.05% Tween-20) for 1 h and probed with primary antibodies against: NF-κB p50, NF-κB p65, Bcl-2, Bax, procaspase-9 p35 (Santa Cruz Biotechnology, Santa Cruz, CA), and GAPDH (Chemicon International). After 3 washes, the membrane was further incubated with secondary antibody conjugated with horseradish peroxidase (HRP). The expression of targeted proteins was detected with the enhanced chemiluminescent (ECL) detection system (Amersham, Buckinghamshire, UK).

### Apoptosis assay

Analysis of apoptosis was performed using Annexin V-FITC Apoptosis Detection KIT I (BD Pharmingen). Briefly, OVA-14 cells were incubated with sulindac (100 μM) and PDTC (16 μM), either alone or in combination, for 4 or 24 h. At the end of the incubation the cells were trypsinized, washed twice with cold PBS and then resuspended in binding buffer at a concentration of 1×10^6^ cells/ml. Cell suspensions (1×10^5^ cells in 100 μl) were transferred to 5-ml tubes, mixed with 5 μl FITC Annexin-V and 5 μl propidium iodide (PI), vortexed, and incubated for 15 min at room temperature in the dark. Next, 400 μl of binding buffer was added to each tube and the samples were measured using a flow cytometer (FACSCalibur; Becton-Dickinson, Mountain View, CA) and CellQuest software.

### Flow cytometric analysis of cell cycle

OVA-14 cells were cultured with sulindac (100 μM) and PDTC (16 μM), either alone or in combination, for 24 h. The cells were then trypsinized, washed twice in PBS and fixed (~1×10^6^) in 3 ml of 70% ethanol (−20°C). After incubation at −20°C for 48 h, cells were washed three times in PBS and stained with 50 μg/ml propidium iodide (PI) and 25 mg/ml RNase in PBS for 30 min in dark at room temperature. The samples were analyzed using a flow cytometer (FACSCalibur) and the CellQuest software.

### Statistical analysis

Data are presented as mean ± standard deviation (SD). Results of MTT assay were analyzed by the Student’s t-test. Additionally, the nature of the interaction between tested drug combinations was analyzed by the method described by Chou and Talalay (33). The combination index (CI) method is a mathematical and quantitative evaluation of a two-drugs pharmacological interaction. Using data from the cytotoxicity experiments and CalcuSyn ver. 2.0 software (Biosoft, Cambridge, UK), CI values were generated. CIs of <1 indicate synergism, CIs=1 indicate additivity, and CIs >1 indicate antagonism.

## Results

### Combinations of sulindac or sulindac sulfide with PDTC synergistically inhibit the growth of OVA-14 cells in vitro

**Figure 1:**
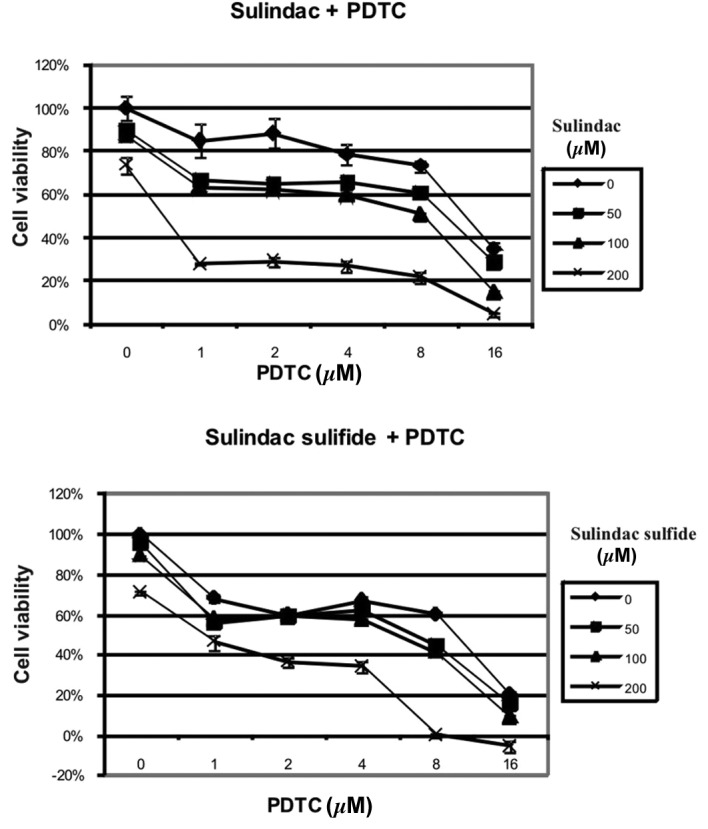
Effect of PDTC and sulindac (A) or sulindac sulfide (B) on viability of OVA-14 ovarian cancer cells. OVA-14 cells were incubated for 24 h with 1, 2, 4, 8 or 16 μM of PDTC in combination with 50, 100 or 200 μM of sulindac or sulindac sulfide. Cell viability was measured by MTT assay. The data show the mean ± SD (n=3). Strong synergistic effect is exhibited especially in cultures incubated with 200 μM sulindac or sulindac sulfide with 16 μM PDTC (CI<0.01 and CI<0.4, respectively).

**Figure 2:**
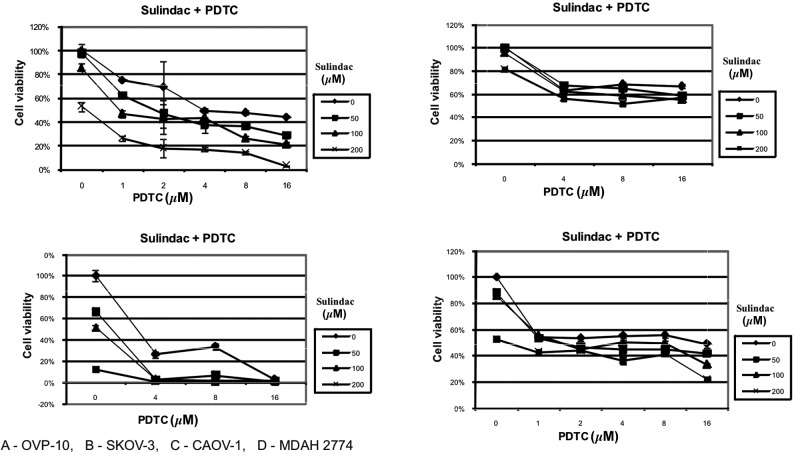
Effect of PDTC and sulindac on the cell viability of different ovarian cancer cell lines: (A) OVP-10, (B) SKOV-3, (C) CAOV-1 and (D) MDAH 2774. The cells were incubated with different concentrations of sulindac and/or PDTC for 24 h and cell viability was measured by MTT assay. The data show the mean ± SD (n=3).

In our preliminary experiments, sulindac and sulindac sulfide were the most effective agents, from amongst other COX-1/COX-2 inhibitors (including acetylsalicylic acid, rofecoxib, and sulindac sulfone), in inhibition of the viability of various ovarian cancer cell lines (32). To determine if effectiveness of these two agents could be enhanced by PDTC we incubated OVA-14 ovarian cancer cells with different concentrations of these compounds for 24 h. As measured in the MTT assay, both sulindac and sulindac sulfide synergized in the cytotoxic effect with PDTC (Fig. 1). For example, incubation with 200 μM sulindac resulted in 72% viability, 16 μM PDTC resulted in 37% viability, and the combination of these two doses of drugs reduced viability of tumor cells below 5% (Fig. 1A) (CI <0.003). The synergistic effect of sulindac and PDTC was also manifested in CAOV-1 and OVP-10 cell cultures. MDAH 2774 and SKOV-3 cells were found to be susceptible to sulindac or PDTC but the combination of these two agents did not result in synergy (Fig. 2).

### The combination of sulindac with PDTC results in cell cycle arrest

**Figure 3:**
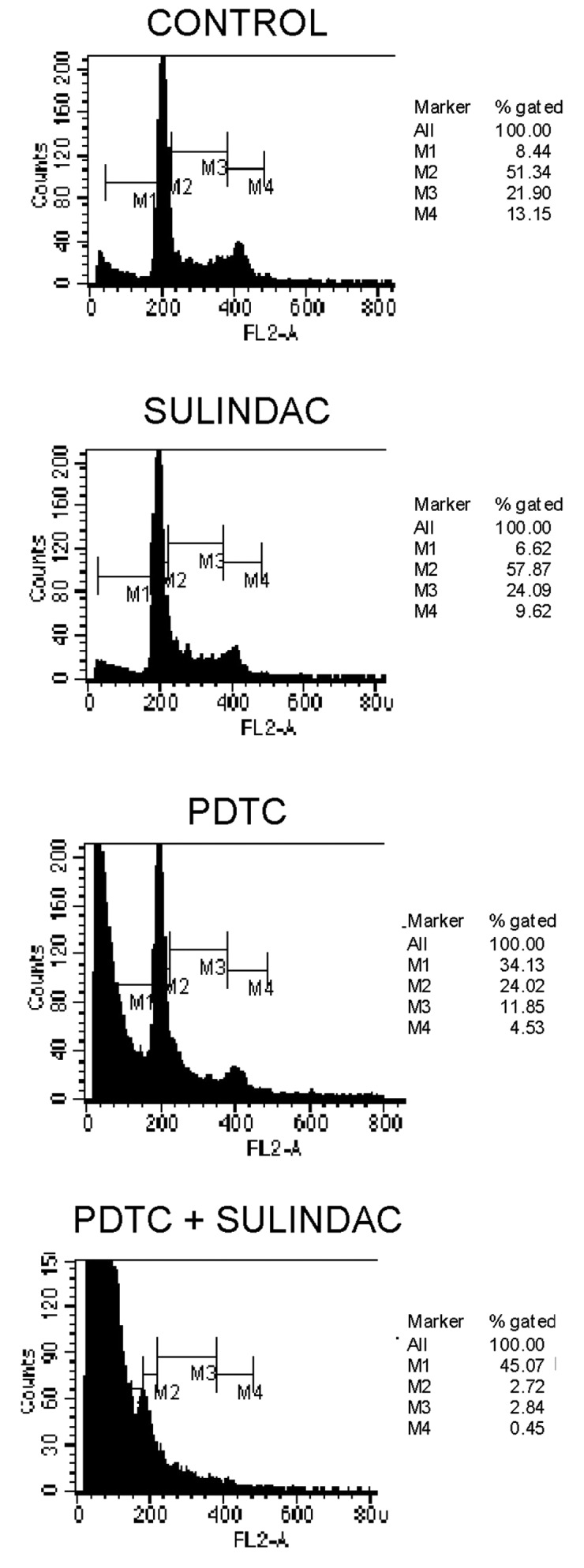
Effect of sulindac and PDTC on the cell cycle in OVA-14 cells. The cells were incubated with 100 μM sulindac and/or 16 μM PDTC for 24 h, fixed in 70% ethanol, stained with propidium iodide, and analyzed for cell cycle distribution by flow cytometry.

To evaluate the effects of sulindac and PDTC on cell cycle behavior, cell cycle analysis by FACS was performed. We found that incubation of OVA-14 cells with 100 μM sulindac did not influence cell cycle progression (Fig. 3). On the other hand, 16 μM PDTC induced inhibition of a cell cycle in the sub-G1 and G0/G1 phases. Combination treatment with sulindac and PDTC, however, resulted in the strongest inhibitory effect and a dramatic increase of cell number in a sub-G1 phase; only 5% of the cells progressed to the G0/G1 + M + G2 phases (Fig. 3).

### The synergistic cytotoxic effect of sulindac and PDTC can be attributed to apoptosis

**Figure 4:**
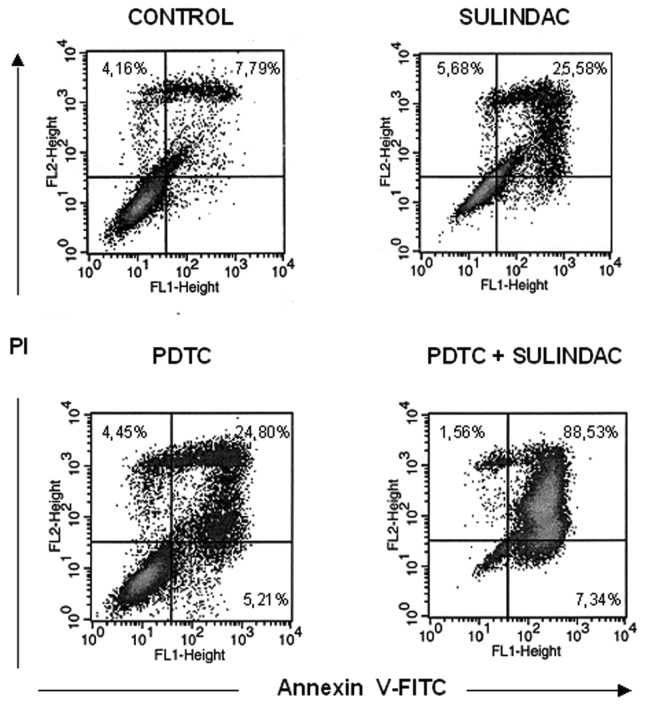
Annexin-V/PI dual staining of OVA-14 cells incubated with 100 μM sulindac and/or 16 μM PDTC for 24 h. Numbers express the percentage of cells that were double-stained, single-stained or unstained with Annexin-V and propidium iodide. The lower right quadrant represent early apoptosis and the upper right quadrant (double-stained cells) late apoptosis cells.

**Figure 5:**
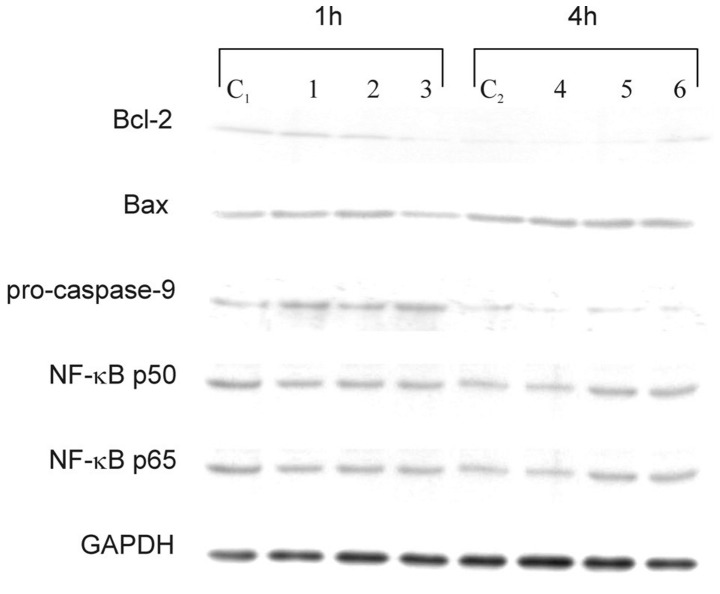
Effect of sulindac and PDTC on expression of Bcl-2, Bax, procaspase-9, NF-κB p50 and NF-κB p65 in OVA-14 cells incubated with 100 μM sulindac and/or 16 μM PDTC for 1 or 4 h. Cytoplasmic cell lysates were immunoblotted with antibodies against each of the above-mentioned proteins, as described in Materials and methods. GAPDH was used as control. C1, C2, controls; lanes 1 and 4, cells incubated with sulindac alone; lanes 2, 5, cells incubated with PDTC alone; lanes 3 and 6, cells incubated with both sulindac and PDTC.

To determine if decreased viability of OVA-14 cells in cultures with sulindac and PDTC result from apoptosis or other mechanisms, the cells were incubated with 100 μM sulindac and 16 μM PDTC, either alone or in combination for 4 or 24 h, with subsequent Annexin-V flow cytometric analysis. There was a slight increase in the percentage of cells in early apoptosis [Annexin-V(+), propidium iodide(−) cells] in double-treated cultures in comparison with cells incubated with sulindac alone, PDTC alone or untreated cultures (2.14%, in comparison with 1.49, 1.42, and 1.44%, respectively) at the 4-h incubation timepoint (data not shown). In 24-h cultures, the process of apoptosis progressed and was especially exhibited in OVA-14 cells co-treated with sulindac and PDTC: 89% cells were found to be fully apoptotic, in comparison with 26% in sulindac cultures, 25% in PDTC cultures, and 8% in control cultures (Fig. 4). To shed some light on the intracellular mechanisms responsible for apoptosis progression in the cells co-incubated with sulindac and PDTC we examined, by Western blotting, the expression of various proteins that are characteristic of the apoptosis pathway. Cytoplasimc cell lysate proteins did not show any significant changes in the expression levels of Bax, NF-κB p50, and NF-κB p65, either in cells treated with sulindac and PDTC alone or in combination (Fig. 5). No effects of these two agents on NF-κB and no NF-κB DNA-binding activity were confirmed in an EMSA (data not shown). Western blot analysis revealed a decrease of antiapoptotic Bcl-2 protein after 1 h of incubation of OVA-14 cells with both agents and a drop of procaspase-9 in single- and double-agent-treated cultures (Fig. 5). The latter observation argues for activation of caspase-9, which is typical for the downstream activation of the mitochondrial pathway of apoptosis.

## Discussion

A combination approach of several therapeutic agents has become the standard treatment of ovarian cancer. In the present study, we demonstrated that the combined use of sulindac or sulindac sulfide and PDTC *in vitro* resulted in a synergistic cytotoxic effect against OVA-14 ovarian cancer cells (Fig. 1). This effect was also observed in our studies on other ovarian cancer cell lines (OVP-10, CAOV-1) (Fig. 2). The primary mechanism responsible for the cytotoxic effect of sulindac + PDTC was apoptosis (Fig. 4). Increased level of Bcl-2 and decreased expression of procaspase-9 (reciprocally proportional to the active form of caspase-9) argues for the mitochondrial apoptotic pathway and is in agreement with results of other investigators showing correlation of caspase-9 activation and cancer cell death following incubation with sulindac or sulindac sulfide (16) or PDTC (26). In our model, susceptibility of OVA-14 cells to the cytotoxic effect of sulindac and PDTC was not related to the expression of COX-1/COX-2 since both sulindac sulfide (an active inhibitor of cyclooxygenases *in vitro*) and sulindac (inactive compound) synergized with PDTC. Moreover, OVA-14 cells did not express COX-1 and COX-2 enzymes in our preliminary experiments (data not shown).

Both sulindac and PDTC have been shown to inhibit the pro-survival NF-κB signaling pathway in different tumor models (29,34–36). However, interfering with the NF-κB pathway did not seem to play a role in the cytotoxic effect of sulindac and PDTC in our studies (Fig. 5). This observation does not exclude the possibility of NF-κB inhibition by sulindac and/or PDTC in case of activation of NF-κB in some circumstances, e.g. following standard chemotherapy (30) or in tumor cells with high constitutive expression of NF-κB. Recent results of Nai *et al* (37) argue for the latter scenario; they reported that application of PDTC *in vivo* led to a decreased level of NF-κB (and also prevention of cachexia) in tumor tissue in a mouse model of colon cancer.

COX inhibitors, including sulindac and its metabolites, have been recognized as promising agents in the prevention of neoplasia and in cancer treatment, especially when combined with standard therapy [(38) and clinical trial NCT00755976). Several *in vitro* studies have shown their antitumor efficacy in ovarian cancer models (10,13,19,23). On the other hand, PDTC has been reported to inhibit the growth of SKOV-3 ovarian cancer cells and to increase chemosensitivity of these cells to paclitaxel (30). Combination of sulindac and PDTC has not been tested before, to our knowledge, in any tumor model but, as shown in our investigation, their joined application seems very promising.

Apart from the direct pro-apoptotic activity of sulindac and PDTC, which we and others observed *in vitro*, these agents exert pleiotropic biological effects *in vivo*, beneficial from the therapeutic point of view. Upregulation of COX enzymes is a frequent feature of neoplasia and in ovarian cancers COXs have been implicated in platinum resistance and promotion of tumor progression (11,12). Furthermore, taxanes, that are standard drugs used in first- and second-line therapy of ovarian cancer, have been shown to induce COX-2 expression (39). Therefore, one may expect that sulindac may increase the efficacy of these drug classes. Sulindac, alone or in combination with thalidomide, inhibited growth of carcinoma in rabbits (8) and was also effective in suppressing pancreatic tumor growth in a xenograft model in mice (14). Recently, sulindac in combination with epirubicin has been tested in patients with advanced cancers and stabilization of the disease in a patient with ovarian carcinoma was observed (38). Since PDTC, apart from potent NF-κB inhibitory properties and direct cytotoxic effect against tumor cells, manifests strong anti-angiogenic effects (31,40), its addition to sulindac-based therapy could reveal stronger antitumor activity *in vivo* than those observed in ovarian cancer cell cultures *in vitro*. Thus, preclinical *in vivo* investigations of potential antitumor effect of PDTC and sulindac against ovarian cancers are worth continuing.
